# A deterministic approach for rapid identification of the critical links in networks

**DOI:** 10.1371/journal.pone.0219658

**Published:** 2019-07-17

**Authors:** Rostislav Vodák, Michal Bíl, Tomáš Svoboda, Zuzana Křivánková, Jan Kubeček, Tomáš Rebok, Petr Hliněný

**Affiliations:** 1 CDV–Transport Research Centre, Brno, Czech Republic; 2 Faculty of Science, Palacký University, Olomouc, Czech Republic; 3 CESNET, Prague, Czech Republic; 4 CERIT-SC, Institute of Computer Science, Masaryk University, Brno, Czech Republic; 5 Faculty of Informatics, Masaryk University, Brno, Czech Republic; University of Sydney, AUSTRALIA

## Abstract

We introduce a rapid deterministic algorithm for identification of the most critical links which are capable of causing network disruptions. The algorithm is based on searching for the shortest cycles in the network and provides a significant time improvement compared with a common brute-force algorithm which scans the entire network. We used a simple measure, based on standard deviation, as a vulnerability measure. It takes into account the importance of nodes in particular network components. We demonstrate this approach on a real network with 734 nodes and 990 links. We found the worst scenarios for the cases with and without people living in the nodes. The evaluation of all network breakups can provide transportation planners and administrators with plenty of data for further statistical analyses. The presented approach provides an alternative approach to the recent research assessing the impacts of simultaneous interruptions of multiple links.

## Introduction

Modern society is highly dependent on various types of networks, among which road networks occupy the most prominent place. People would not be able to utilize even the most basic services, such as medical care, without a functioning road network. An efficient road network thus ranks among the priorities for any society. Its serviceability can be affected, however, by various types of events which originate within the transport system (such as traffic accidents, congestions, technical failures, etc.) but also by events caused by external forces (such as floods, landslides, heavy snowfalls, storms, wildfires, earthquakes, etc.). The most challenging issue for road administrators is the development of methods which can help in dealing with and preventing critical situations when both types of events occur.

Identification of critical road links is part of vulnerability analysis of transportation networks. This analysis pays attention to particular links and evaluates their importance within the whole network. The manner in which reduced capacity of a link or its complete blockage will affect the functioning of the entire network is often studied. A large number of road links are sometimes interrupted concurrently for various reasons. Such a situation can lead to cascading effects when other links collapse and the overall impact on the network performance is enormous. It is therefore important to analyze impacts of as many as possible combinations of concurrently interrupted links. Such an analysis is, however, computationally demanding. It requires the application of additional restrictions on the set of analyzed links. These restrictions often encompass certain properties which are common for the set of the links in question. This means, for instance, that the links are located in the same region and are therefore close to one another.

In this paper, we focus our attention on disasters when the road links are completely interrupted and a road network is disintegrated into several isolated parts. Such a situation can result in a number of people cut-off from sources of food, water and medical treatment. When such events occur, a rescue effort related to the reconnection of isolated components with a high number of people has the highest priority. We thus introduce a simple but practical measure evaluating network disintegration based on the overall number of people isolated from the primary network.

We introduce, in this work, a novel deterministic algorithm, based on cycles in graphs, which enables the identification of the most critical links and reduces computational demands. The suggested algorithm thus identifies all possible road network break-ups caused by up to 9 concurrently interrupted links. Identification of all the decompositions of the network, for the defined number of interrupted links and their evaluation, is the aim of this work.

## Literature review

The importance of networks in everyday life leads to the need to study their properties. Serviceability, accessibility and vulnerability rank among the most prominent concepts at present [1–5]. In this paper we pay attention to vulnerability. The first definition of vulnerability in the road transportation system is based upon a susceptibility to events (incidents) that result in considerable reductions in road network serviceability [6]. From the definition it follows that vulnerability includes probabilities of individual road link interruption, by, e.g., landsliding [7–9], capacity reduction [10] and demand variation [11]. It should be pointed out that an approach based upon such probabilities requires a sufficient amount of data which may not always be available [10]. This is specifically valid for extreme natural events which are rare [12]. We also refer the reader to [13] for the development of link failure duration probability distribution based upon the Monte Carlo simulation method. In these situations, the second definition of vulnerability, which does not require any specific value of probability, comes into play. Throughout the paper, we use the second definition which means the identification and evaluation of such combination of road links whose disruption has the largest negative impact on the functioning of the network despite the low probability of such an event [6]. The usefulness of the study of the problem was pointed out in [2] (see also [14,15]). We call the links which correspond to the worst cases *critical*. The concept of criticality corresponds to the concept of importance developed in [14–16]. We thus follow the path where the reliability and the vulnerability of the network are related to its connectivity [17,18] and where any combination of links should be studied [19,20]. The problem is also important for networks of any size [16]. For further discussion concerning the terms we refer the reader to [1] and [21].

The first issue is how to evaluate various combinations of interrupted road links. One can draw inspiration from the vast source of literature covering vulnerability measures. The work [22] provides a solid starting point as they present tests of several measures. We also refer the reader to [23] and [6] for a comprehensive review about vulnerability. A discussion about the connection between reliability and vulnerability together with an analysis of several indices can be found in [24]. The vulnerability measures can be related to numerous things which are of importance for users and/or for the network administrators. In [25], the authors used four centrality indices to analyze the network structure. [14] suggest measures based upon travel costs and unsatisfied demands. The measures can be understood as a generalization of the concepts based upon travel-time costs and travel time [26,27]. Additional important measures are based upon accessibility or *s-t* path availability [2,28,29]. By the *s-t path* we mean the shortest path connecting nodes *s* and *t*. A simple but effective measure uses the shortest paths among the nodes [30]. Another network vulnerability index based upon the impact area was introduced in [19]. If we do not know whether the road links will be interrupted or only partially damaged, we can use methods comprising the capacity reduction approach [31,32] or the macroscopic fundamental diagram [16]. A recent paper [33] draws attention to the accessibility of emergency services and can be applied to a disintegrated network as well. The advantage of most of the above-mentioned measures is that they can be applied to worst-case scenarios to improve the resilience of a network [34,35]. An interesting approach to the network vulnerability is based upon the game theory [36,37]. A number of studies can also be found on road network performance in terms of vulnerability and robustness [2,3,14,15,26–28,31,38–45]. The recent book [46] reviews the range of existing approaches to network vulnerability with their application to transport networks.

Another issue is the identification of the worst-case scenarios. This is difficult, however, to solve due to high computational demands. For instance, if a network consists of 1,000 road links and we plan to evaluate all combinations for 3 concurrently interrupted links, we have to process 166,167,000 combinations. The number of combinations rises to 41,417,124,750 for 4 links. These numbers suggest that the respective state space (all possible combinations of interrupted road links) is extremely large and that to evaluate any combination of disrupted road links using a brute-force examination is beyond the scope of current-day computers (see for instance [2] for a brute-force simulation-based approach). This is the reason why, despite the fact that some of the vulnerability measures can be applied to any combination of interrupted road links, many of the above papers only pay attention to one affected link. Other papers try to reduce the burdensome computation by pre-selecting potential vulnerable links [47,48] or by reducing the area influencing vulnerability index [19]. Only a limited number of works cover the case with 2 or more *concurrently* interrupted links. These papers are mostly devoted to analyses of the impacts of natural disasters [3,49–51]. Apart from the paper [51], the above-mentioned papers compared pre- and post-catastrophic scenarios and measured their impacts. [51] analyzes several scenarios caused by a small number of interrupted links. These links were, however, restricted to a fairly small area given by a predefined grid representing the extent of natural disaster. The approach excludes, however, many events including natural disasters [12]. The scenarios do not take into account, for instance, the simultaneous occurrence of other events such as traffic accidents which can cause the road link disruption (or blockage) in other parts of the network. In addition, links whose interruption can cause the largest damage to the network need not be found in the same area but can be distributed within the network [12]. Certain links (or in some special cases nodes) can then, for example, be the target of a terrorist attack [52,53]. One of the first attempts in the area is the paper [20], where attention was paid to identification of vulnerable links in transportation networks. The vulnerability is measured by total travel costs and the problem is formulated as a mixed-integer nonlinear problem with equilibrium constraints. The approach is demonstrated on small networks with combinations of up to three interrupted links. The links causing the disintegration of the network are, however, excluded. The idea of the approach is to identify critical links without the need to explore all combinations of links. The idea is further developed in [54], where the upper and lower bounds of transportation network vulnerability are obtained using a binary integer bi-level program. The approach also includes the use of the virtual link capacity-based maximum flow and the virtual link cast-based shortest paths problem formulation. The developed mathematical model must be further linearized in order to be solved by commercial software. The results are demonstrated on very small network (6 nodes and 16 links) but time of the computation is not provided. Further approaches based upon the game theoretic approach and sensitivity and uncertainty analyses can be found in [55–57] and [21,58,59], respectively.

In this paper, attention is thus paid to network disintegration into several parts caused by concurrent interruption of several road links. This issue of finding the most critical links is related to the problem of generating the partitions of a graph into a fixed number of cuts evaluated by a function. A *cut* (or *cut-set*) in a graph is defined in graph theory as a set of links partitioning the graph into two disjoint node subsets. End nodes of the links in the cut are in the different subsets. A *minimum cut* of a graph is a cut *of minimum total weight*. In the case of the disintegration of the network into more than two components, we can similarly introduce a *k-cut* and a *minimum k-cut* of a graph. For a brief review about recent developments in this field, we refer the reader to [60] for the minimum 3-cut problem, [61] for the minimum 3- and 4-cut problem and [62] for the minimum 5- and 6-cut problem. Further deterministic algorithms, based upon the cactus representation or maximum flow computation, can be found in [63–65]. In case of an interest in network disintegrations for a given number of links, the approach based on minimum k-cuts is not applicable because in many road networks it would lead to finding only the nodes with one link connecting them to the rest of the network. Moreover, according to the above papers, it seems that the techniques developed for minimum 3-cuts and 4-cuts are not simply extensible for higher cuts and are not suitable for minimization of a loss function. Another related problems are so-called *(s*, *t)-cuts*, i.e., the cuts which contain nodes *s* and *t* in different components, and their generalizations *(S*, *T)-cuts* for a set of nodes *S* and *T* [66]. Another interesting approach is based on spectral analysis. It requires computation of the second eigenvalue of the graph Laplacian. The method provides information on how the particular clusters are connected in the network [67,68]. The approach enables the identification of bottlenecks in the network if the capacities of the links are known [5]. For further applications of this method we refer to [69,70].

In our algorithm we do not take into account any flows in the network (see for instance [71–74]) because they are dramatically changed during many events with a larger number of interrupted links. The common traffic pattern [75–78] completely disappears as well and the standard traffic control begins to be useless. The situation is also much more serious than the common congestions [79] and the transportation system is far out of its equilibrium [80]. In addition, the measure, which we introduce in this paper, also enables the reader to easily verify the results. On the other hand, more sophisticated measures working with flows, demands and supplies information can be useful in many break-ups with a limited influence on the entire network and can be part of the analysis based upon our algorithm.

## Methods

In this section, a network vulnerability measure, represented by a suitable loss function, is introduced along with a new rapid and efficient deterministic algorithm.

**Fig 1:**
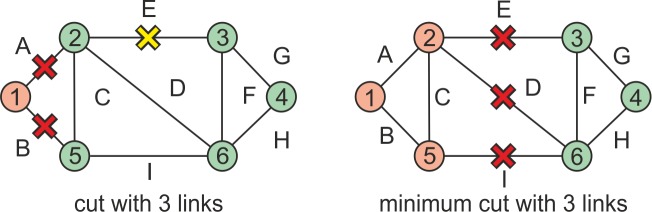
An example of a cut and minimum cut for 3 interrupted links.

For the purpose of this paper we modified the definitions of cuts and minimum cuts. By a *cut* we mean any set of links whose concurrent interruption leads to the disintegration of the network into two parts. It should be pointed out that it is not necessary for all the links to participate in the disintegration, i.e., if some of the links are made passable, there is still a cut. By a *minimum cut* we mean a cut where *all* the links participate in the disintegration, i.e., if any of the links is made passable, no cut exists. The definitions of *k-cuts* and *minimum k-cuts* are then similar. The difference between a *k-cut* and a *minimum k-cut* is demonstrated in Fig 1.

### Algorithm

The proposed deterministic algorithm makes possible finding all disintegrations of a network for a given number of links in a reasonable time without complete examination of the large state space of the road network. This algorithm is able to examine all minimum k-cuts of the given network under the predefined numbers of cut-set links and components or further limitations.

To describe the algorithm, we use the standard notation in graph theory, namely *G* = (*V*,*E*) denotes the *graph* representing the road network, where *V* is a set containing all nodes and *E* is a set of all links. By a *closed walk* we mean the sequence of nodes and links

$$W=v_{0},e_{1},v_{1},e_{2},\ldots ,e_{n},v_{n},$$

where *v*_*i*_∈*V*, *e*_*j*_∈*E* and *v*_0_ = *v*_*n*_. A *cycle* is a closed walk with at least three links where the nodes and links appear only once except for the first and the last node.

The idea of the algorithm is as follows. Assume there is a link and its ending nodes. If a cycle containing the link exists for the nodes, the nodes belong to the same component. Otherwise, they belong to different components. Part of the cycle then indicates a detour. To the best of our knowledge, no algorithm based upon searching for cycles (see below for a more detailed description) has ever been used for an analysis of a disintegrated network.

We now provide a precise description of the algorithm using the following pseudocode. A *spanning tree T* of an undirected graph *G* is a special form of a subgraph with minimum possible number of links and all the nodes of *G*. The transformation of the graph into a spanning tree is not a key part of our algorithm but enables to reduce the number of links in step 1 which the algorithm has to go through.

Input conditions:

*graph* – Connected graph

*maxLinks* – Integer number within interval [1, |*E*|] denotes the maximum number of links in the found cut-sets.

*maxComponents* – Integer number within interval [2, *maxLinkss*+1] denotes the maximum number of components generated by the found cuts.

**Algorithm 1:** *findMinimalCutSets*(*graph*,*maxLinks*,*maxComponents*)

// initialization

**global** *graph*,*maxLinks*,*maxComponents*

**global** *spanningTree* = *getSpanningTree*(*graph*)

*CS* = ∅

// processing

**for every link** *link*∈*spanningTree* **do**

    *CS* = *CS*∪*findCutSets*(1,∅,*link*)

**endfor**

// filtering not minimal cut-sets

*minCS* = ∅

**for every cut-set** *cs*∈*CS* **do**

    **if** *isMinimalCutSet*(*cs*) **do**

        *minCS* = *minCS*∪{*cs*}

      **endif**

**endfor**

**return** *minCS*

**Algorithm 2:** *findCutSets*(*level*,*restrictedLinks*,*link*)

*restrictedLinks* = *restrictedLinks*∪*link*

*foundCS* = ∅

*cycle* = *findShortCycle*(*link*,*restrictedLinks*)

**if** *cycle* exists **then**

    **if** |*restrictedLinks*|<*maxLinks* **then**

        **for every link** *c*∈*cycle* **do**

            *foundCS* = *foundCS*∪*findCutSets*(*level*, *restrictedLinks*,*c*)

        **endfor**

    **endif**

**else**

    *foundCS* = *foundCS*∪{*restrictedLinks*}

    **if** |*restrictedLinks*|<*maxLinks AND level*+1<*maxComponents* **do**

        **for every link** *f*∈*spanningTree*\*restrictedLinks* **do**

            *foundCS* = *foundCS*∪*findCutSets*(*level*+1,*restrictedLinks*,*f*)

        **endfor**

    **endif**

**endif**

**return** *foundCS*

**Algorithm 3:** *findShortCycle*(*link*,*restrictedLinks*)

*E*′ = *E*_*graph*_\*restrictedLinks*

*G*′ = (*V*_*graph*_,*E*′)

*// node v*_*1*_ *and v*_*2*_ *are both nodes of the link*

$node\;v_{1}={link}_{v_{1}},\;node\;v_{2}={link}_{v_{2}}$

*path* = *findShortPath*(*G*′,*v*_1_,*v*_2_)

**return** *path*

Algorithm 1 begins by determining all the cuts incorporating the links belonging to the graph’s spanning tree (since all the cuts have to incorporate a spanning-tree link). The cuts generated by more links (and containing the particular *link*) are then found by recursive calls of the *findCutSets (level*, *restrictedLinks*, *link)* function (see Algorithm 2), which employs the graph cycles to identify the links belonging to the searched cuts (since every link of a cut has to reduce the number of cycles containing the *link* in the graph). If such a cycle is not available, a cut-set determined by the *restrictedLinks* set has been found and the algorithm may continue by looking for the cuts disjointing the graph into more components. Once all the cuts are found, all the non-minimal cut-sets are filtered out (see the end part of the Algorithm 1). Finally, we describe two auxiliary functions: the function *findShortPath(G’*, *v*_*1*_, *v*_*2*_*)* (see Algorithm 3) finds a path between two nodes *v*_1_,*v*_2_ using a graph algorithm which finds the shortest path in the graph *G*′ (we employ the breadth-first search algorithm), while the function *getSpanningTreeLinks(Graph)* computes the minimum spanning tree in graph *G* and returns a subset of links from *E* which form the minimum spanning tree (employing Kruskal's algorithm for this purpose). A significant advantage of the algorithm is that it can be easily prepared for a parallel implementation, because more computational threads can concurrently process the links stored in the *spanningTree* set (Algorithm 1).

**Fig 2:**
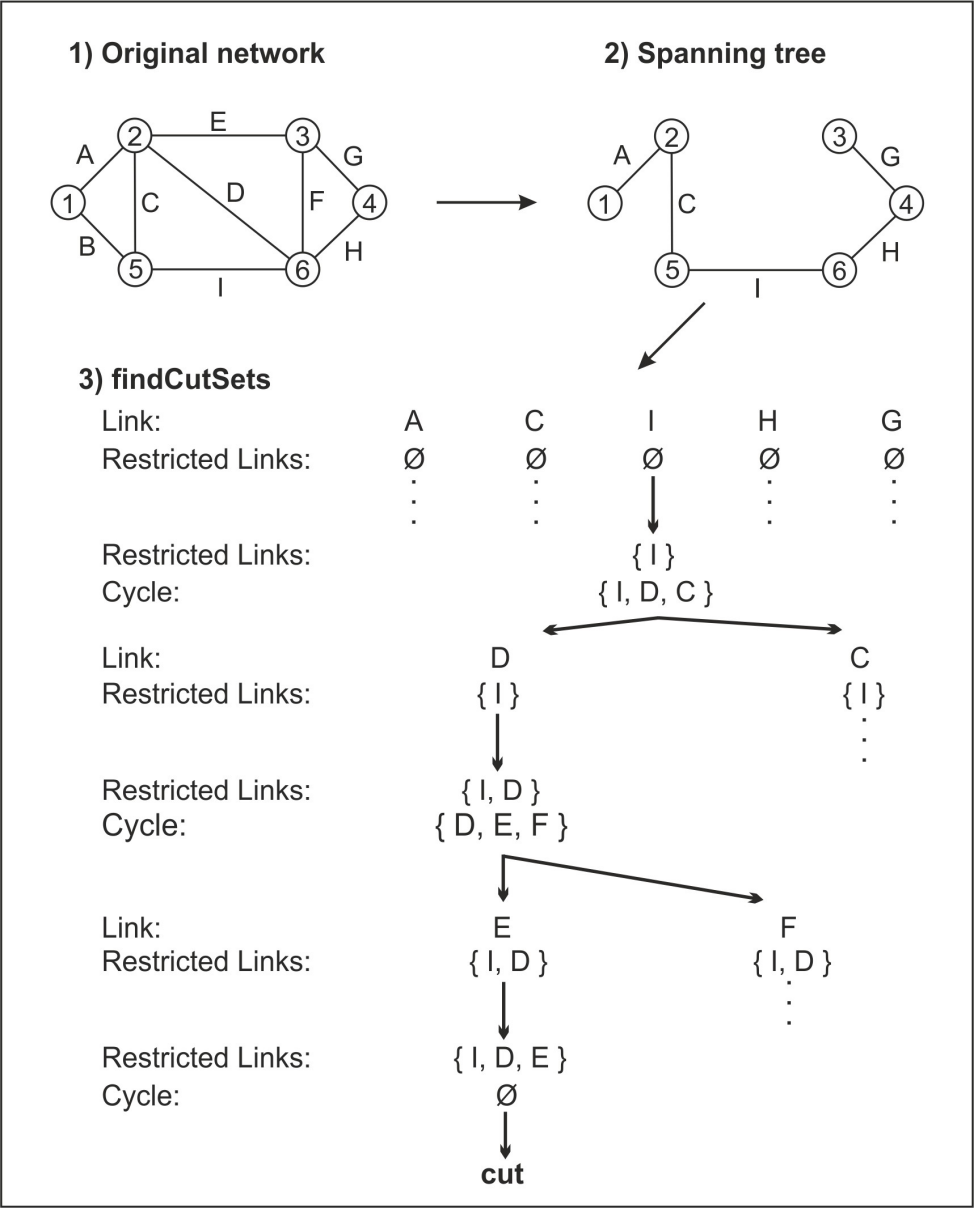
The process of identification of a cut.

Fig 2 demonstrates how the algorithm works on a small network.

**Fig 3:**
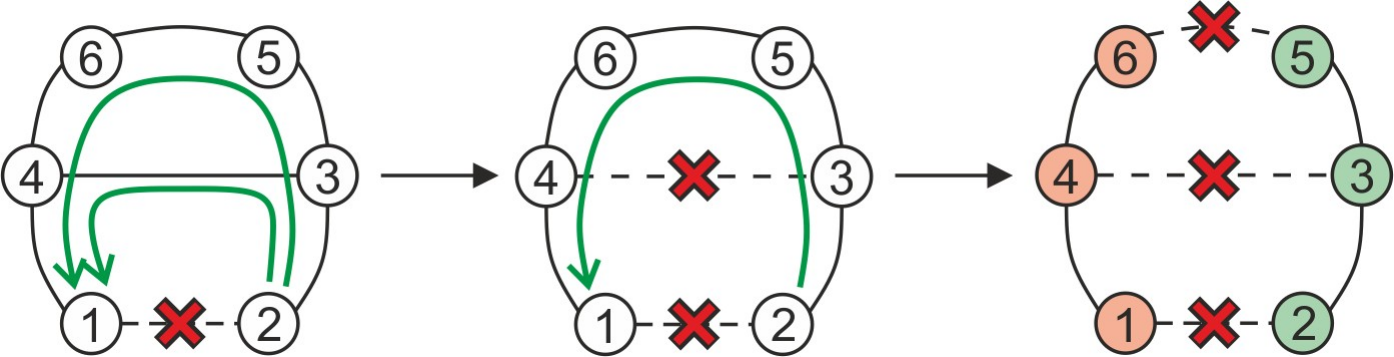
An example of cycles and their interruptions.

In the beginning of the process, a spanning tree of the original graph is generated. Since, in general, a graph may have several spanning trees, any of them can be used for the computation. There is a need to examine all the spanning tree links. One by one, links are inserted into the set of restricted links. This step is illustrated with link *I* in Fig 2. The shortest path between the ending nodes of the link is found, different than just link *I*. The found path together with the link forms a cycle. In the illustrative case from Fig 2, the cycle is {*I*, *D*, *C*}. In the next step, a link from the shortest path is added to the restricted links set. Again, the shortest path is found between the ending nodes of the newly added link. This time, the path must not contain any of the links in the restricted links set {*I*, *D}*. For link *D*, the cycle {*D*, *E*, *F*} is found. The process continues by adding another link to the restricted links set, until no path between its nodes exists. At this moment, a cut-set is identified, which is identical with the set of restricted links {*I*, *D*, *E*}.It is further demonstrated that the algorithm actually finds all the minimum k-cuts for the given number of interrupted links. It is based on the proof for minimum cuts, i.e., the disintegration of a graph into two components. The characterization of the disintegration is the non-existence of any path among the nodes in different components. This means that if two nodes in different components were originally connected with a link, there is no alternative path between them without the link. On the other hand, if two nodes lie in the same component, there is an alternative path which together with the link forms a cycle. This does not apply for dead-end links, however, where the cut appears right after interrupting the link. When interrupting the links on cycles, we thus interrupt alternative paths between the nodes. It is apparent in Fig 3 that by cutting the links on the cycles repeatedly, not only alternative paths for nodes 3 and 4 are interrupted, but also for nodes 1 and 2, etc.

In the end, a minimum cut appears because no alternative path exists for any of the nodes (nodes 1 and 2 in Fig 3). A cut which is not minimal can appear if, for instance, while searching for all disintegrations caused by 5 interrupted links, the disintegration is caused only by 3 of the interrupted links and none of the remaining two links causes separation of another component.

The algorithm is applied recursively on the components which appear after the first run, in order to obtain the minimum k-cuts.

### Measure of network vulnerability

Our primary focus is on such events where a road network breaks up into several *isolated* parts. We are therefore interested, in the first instance, in the number of people cut off from the main network. These people can also be cut off from basic resources such as food, water and medical treatment. It is natural in this case to expect that the worst-case scenario is represented by the state of the network after its disintegration into the maximum number of components with the same number of people living within them. This means that the disintegration of the network into 3 components with the same number of people is worse than the disintegration into 2 components with the same number of people. This is due to the fact that in the first case there is a need to ensure two entries into the components without resources. The suitable loss function representing this vulnerability measure can be defined as follows:

$$F(G_{m})=\sqrt{\frac{\sum_{i=1}^{m+1}(P_{i}-\langle P\rangle {)}^{2}}{m}}$$ (1)

where *m* is the given number of interrupted links, *m+1* is thus the maximal number of components which the network can disintegrate into, *G*_*m*_ is a graph with *m* disrupted links, *P*_*i*_ is the number of people living with the *i*-th component and

$$\langle P\rangle =\frac{1}{m+1}\sum_{i=1}^{m+1}P_{i}.$$

If there are only *m’*, *m’* < *m+1*, components, then *P*_*i*_ = 0 for *i* > *m’*. It is apparent that the defined loss function satisfies the above-mentioned requirements.

The values of *P*_*i*_,*i* = 1,…,*m*+1, need not only represent the number of people. If we put *P*_*i*_ equal to the number of nodes in *i*-th component, we obtain the disintegration of the network into the components with ideally the same number of nodes which relates to the problem of graph partitioning [81]. *P*_*i*_ can also represent the demand or more generally the importance of the *i*-th component.

This is not, however, the only way to define the vulnerability measure. The measures in other papers can be used as well or new measures can be developed based upon the requirements of the contracting authorities. The measures affect the total time of computation but are not incorporated into the algorithm. The process of evaluation of minimum k-cuts proceeds as follows (assume *m* interrupted links):

1. Put *j* = 2
2. If *j*≤*m*+1 compute all minimum j*-*cuts and evaluate them using (1). The information about the number of people in the particular components is found using the breadth-first search. Put *j* = *j*+ 1 and repeat the step. Stop otherwise.
3. Order all evaluated minimum k-cuts.

### Analysis of performance of the algorithm

In this section, the performance of the algorithm on the real network of the Zlín region, which consists of 990 links and 734 nodes, is demonstrated.

It is apparent that there is no possibility to evaluate all the combinations for larger number of concurrently interrupted links. For many networks, the number of break-ups is, however, much smaller. Table 1 provides a comparison of the number of break-ups and the number of combinations of interrupted links.

**Table 1:** The state space for the Zlín region.

Number of concurrently interrupted links	Number of break-ups	Number of all combinations of links	Ratio
1	143*	990	0.14000
2	10,376	489,555	0.02000
3	510,220	161,226,780	0.00300
4	19,154,308	39,782,707,965	0.00048

* This number indicates all dead-end links

The primary contribution of this paper is a proposal of a novel algorithm which is able to efficiently find and evaluate network cuts with a predefined number of concurrently interrupted network links. The principal difference between the proposed algorithm and the brute-force approach is the speed of the computation. Table 2 provides a comparison of the brute-force algorithm with the algorithm employing the cycles approach (during the tests we used 16 CPUs Intel Xeon E7 2.27 GHz, 150 GB RAM).

**Table 2:** Duration of the computation for the Zlín region.

Number of concurrently interrupted links	Brute-force algorithm	Algorithm–Cycles
1	1 s	1 s
2	30 s	14 s
3	10.25 hrs	4 min
4	105 days	11.5 hrs

The shaded table cell represents an estimation of the expected running time of the brute-force approach determining all the cuts generated by 4 interrupted links as it was impossible to measure it precisely. To compute the estimation, we use the number of combinations of 3 and 4 links and the computational time for 3 links. It is apparent that it is impossible to evaluate the scenarios with more than 3 concurrently interrupted links using the brute-force approach. In addition, the Zlín region ranks among the smallest ones in the Czech Republic (only approximately 1,000 road links) and therefore computation of the same scenario for larger regions is not possible.

## Results

In this section, we present the results from the application of the proposed algorithm under various limitations for the Zlín region (Czech Republic) with a population of 587,624 people.

### Disintegration with limitations on the number of components and interrupted links limited to the internal subnetwork

This section examines the internal disintegration of the network since actual networks are usually parts of a larger network (e.g., a network of a region is connected at its borders to the network of the entire country). In order to prevent access to the isolated parts from neighboring regions, we only admit interruption of internal links, i.e., the links which do not lie on the borders of the particular region.

All links which have to be open, in order to study the internal subnetwork, are marked. The algorithm then omits all results related to the combinations of links containing at least one of the marked links.

**Fig 4:**
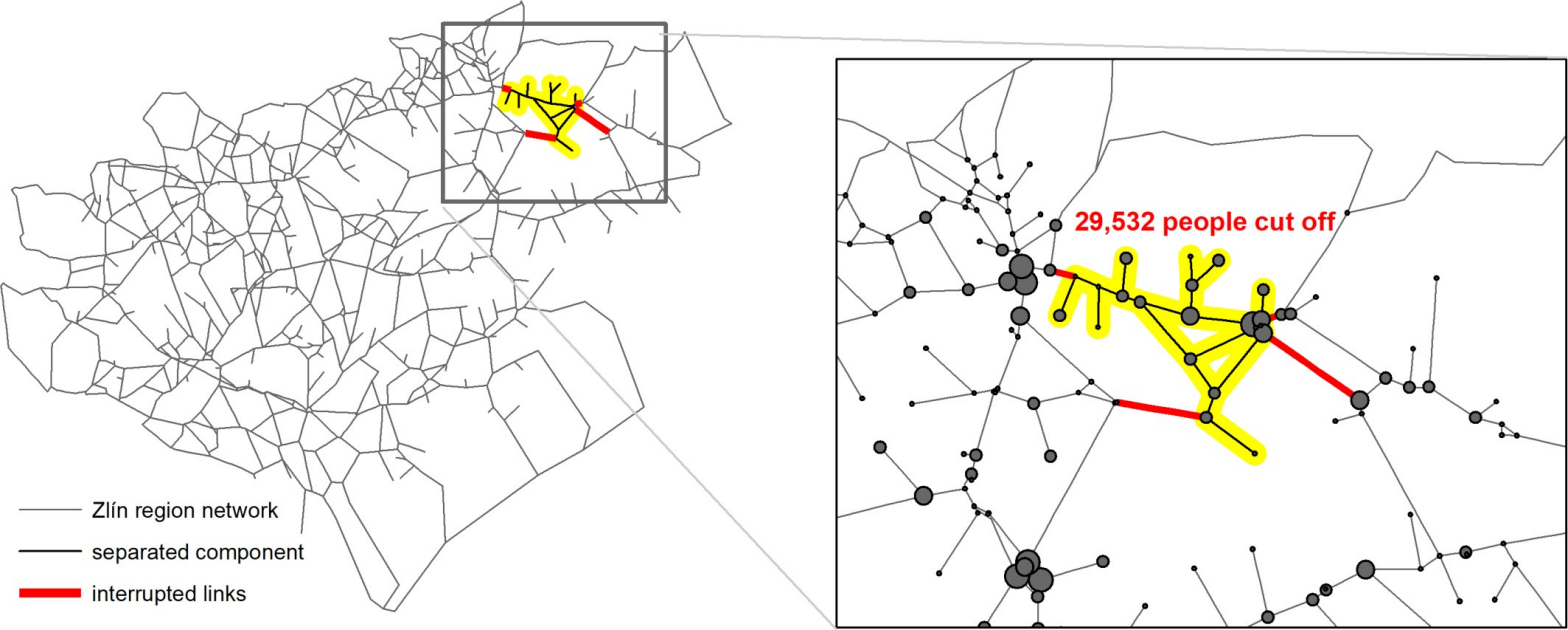
The second-worst-case scenario for the Zlín region. A node diameter represents proportionally the number of inhabitants. The worst scenario is not shown here because it is represented by only one cut-off node, the center of the city of Zlín. This case is more illustrative.

In the example we restrict our attention to the disintegrations of up to 5 components caused by up to 4 links in the Zlín region which seemed to be more interesting than other cases. The results are summarized in Table 3. In Fig 4 we present the case which we consider most interesting, i.e., which do not contain only a node with the largest population and the rest of the network.

**Table 3:** The worst-case scenarios for the Zlín region.

Rank	Number of components	Value of the loss function	Inhabitants in components	Ratio of inhabitants cut off from the main component
1	2	245,391	555,800; 31,824	5.4%
2	2	246,616	558,092; 29,532	5.0%
3	2	247,323	559,410; 28,214	4.8%
4	3	247,384	559,779; 21,511; 6,334	4.7%
5	2	247,592	559,910; 27,714	4.7%
6	2	247,594	559,914; 27,710	4.7%
7	2	247,618	559,958; 27,666	4.7%
8	3	247,800	560,543; 20,747; 6,334	4.6%
9	2	248,280	561,190; 26,434	4.5%
10	2	248,301	561,228; 26,396	4.5%

Fig 4 represents the second worst-case scenario with 29,532 cut-off people (5.0%) after the interruption of four links. The network disintegrates in this case into two components. It is apparent that the case would be difficult to find without computers because it is not concentrated in the area with sparse road network.

### Disintegration with limitations on the number of components, interrupted links and the number of people living in nodes limited to the internal subnetwork

**Fig 5:**
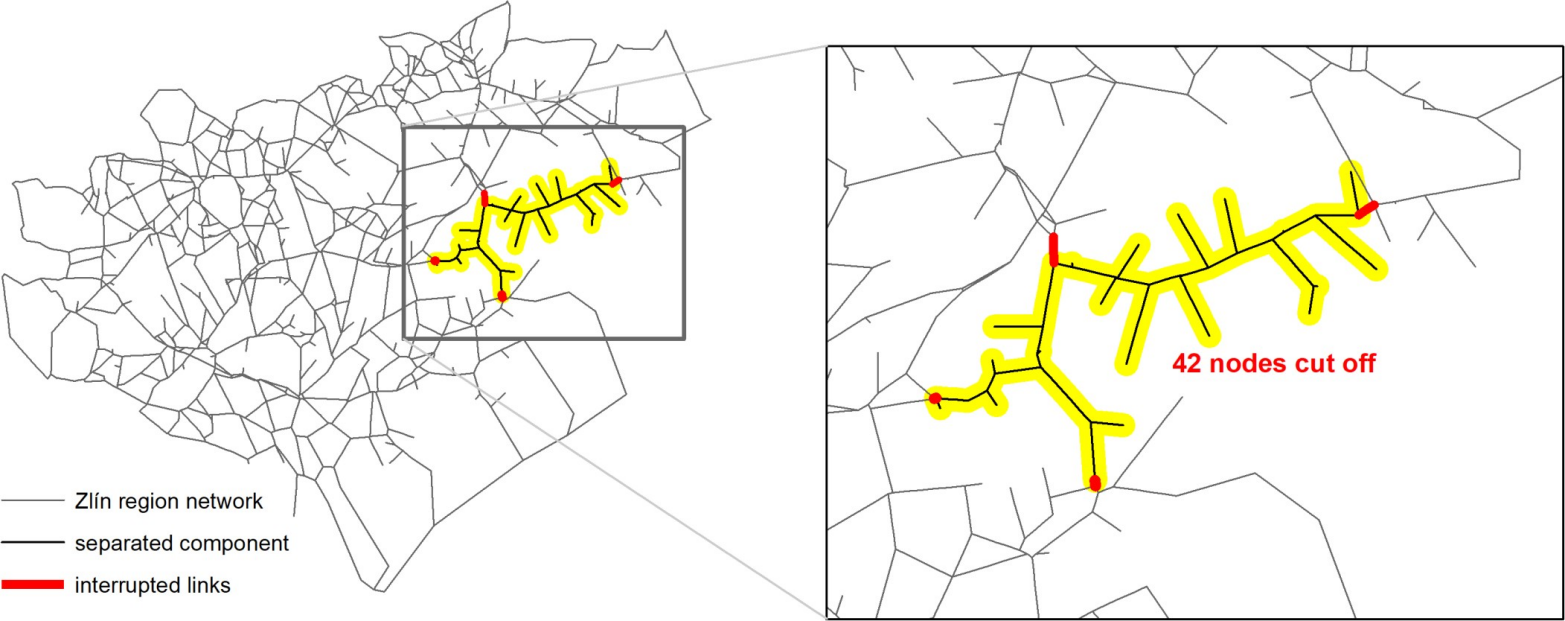
The second-worst-case scenario for the Zlín region. This network disintegration leaves 42 from the 731 nodes (5.7%) out of connection.

This section assumes the same limitations as in the previous one but there is now only one individual living in a node. The results provide us with more information about the spatial structure of the networks than the previous ones. The results concerning the worst-case scenarios can be found in Table 4 and Fig 5 under the requirement of the maximum number of 5 components and 4 links. Only the most interesting case from the visual point of view is depicted here.

**Table 4:** The worst-case scenarios for the Zlín region.

Rank	Number of components	Value of the loss function	Number of nodes in components	Ratio of nodes cut off the main component
1	2	304.8	691; 43	5.9%
2	2	305.3	692; 42	5.7%
3	3	305.8	693; 38; 3	5.6%
4	3	305.8	693; 38; 3	5.6%
5	3	305.8	693; 38; 3	5.6%
6	3	305.8	693; 38; 3	5.6%
7	3	305.8	693; 38; 3	5.6%
8	3	305.8	693; 38; 3	5.6%
9	2	305.9	693; 41	5.6%
10	2	305.9	693; 41	5.6%

If we focus on the number of nodes as inputs into the loss function, the results will look different. The area of the inaccessible part of the network would be larger (see Fig 5) than those only taking into account the number of inhabitants (Fig 4). In this case the results are much more intuitive because they are concentrated in the areas with a sparse road network.

## Discussion and conclusion

As can be seen in the examples above, the algorithm is able to compute the disintegration of the network under various restrictions such as the number of components, the number of interrupted links and the limitation on internal disintegrations. The loss function can also be easily modified because *P*_*i*_ can be understood as weights of components, which measure their importance in the network.

**Fig 6:**
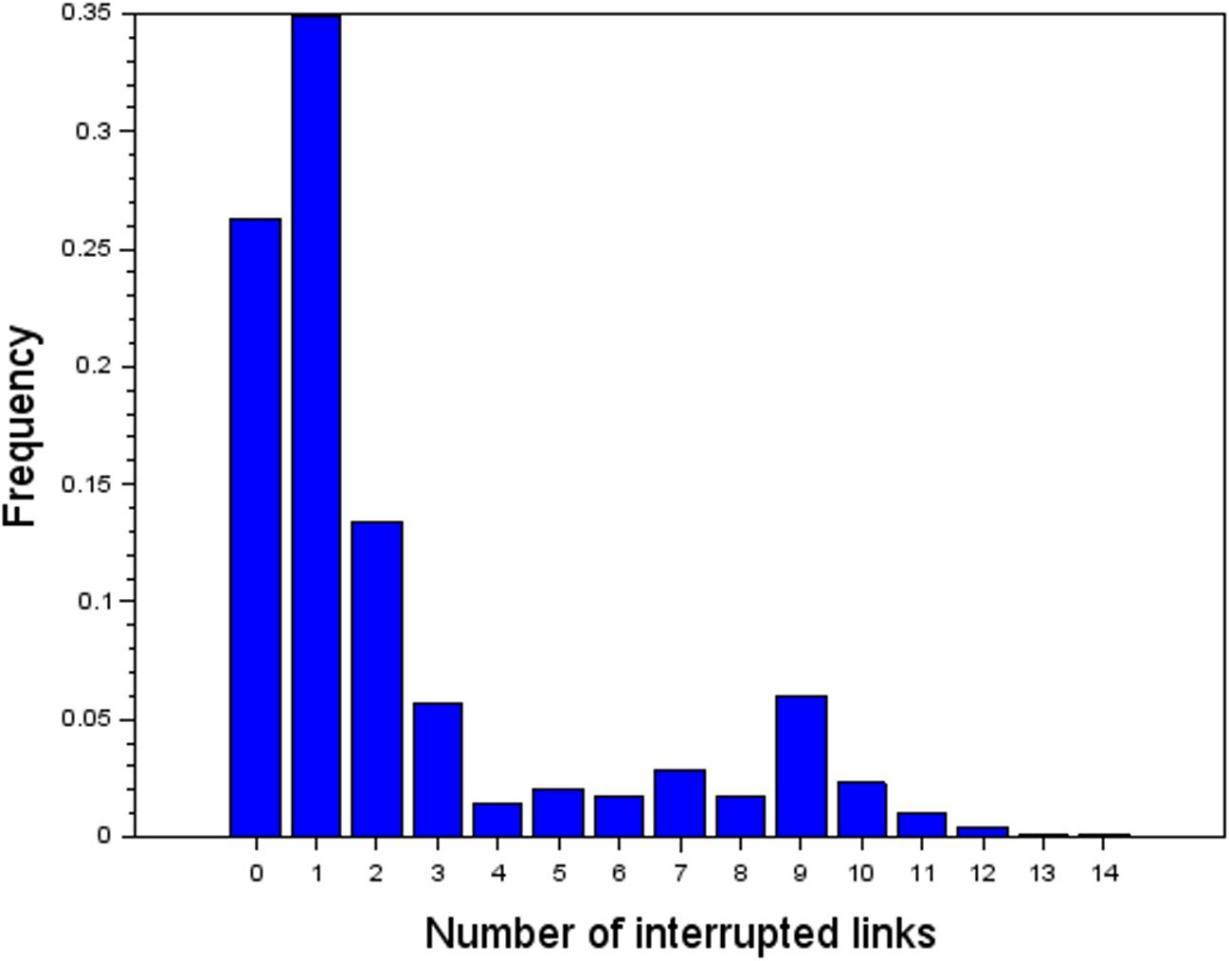
Histogram shows the number of concurrently interrupted links in the Zlín region and their frequencies for 917 days over the years 2014–2016.

To exemplify all the properties of the algorithm and to justify the used number of interrupted links we took data from the Zlín region for 917 days which indicate that the probability of occurrence of a scenario, when four and more road links are concurrently interrupted, is 20% (see Fig 6). These closures were results of various causes, e.g., traffic accidents, planned road maintenance, as well as local road disruptions due to partial flooding.

**Fig 7:**
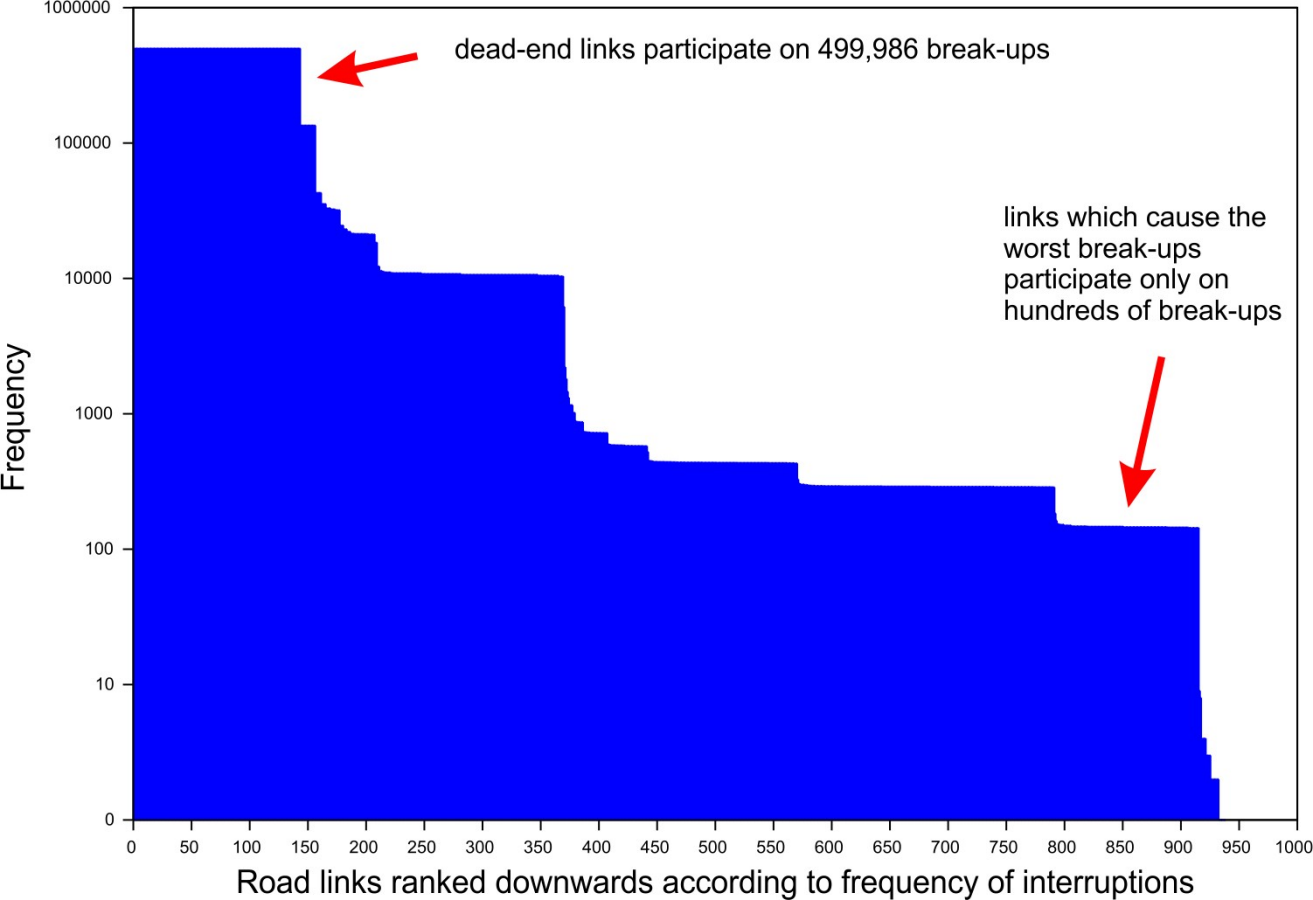
Road links ranked downwards according to frequency of interruptions and the frequency of their occurrence in break-ups. The links which cause the worst break-ups only participate in hundreds of cases.

The vast majority (87%) of break-up scenarios are caused by interruptions of dead-end links and their combinations. The algorithm is also able, however, to find combinations of the links, which are only involved in hundreds of break-ups (see Fig 7).

To demonstrate the necessity of a new fast algorithm we indicated the comparison of the computation time of our algorithm and the brute-force algorithm. As we have presented, the algorithm represents a significant improvement in the computation of network disintegrations. The proposed algorithm was able to compute all the break-ups over 11.5 hours compared with 105 days for the brute-force algorithm (see Table 3).

Despite the ability of the algorithm to noticeably reduce the state space, it nevertheless has to analyze a vast number of combinations which have to be evaluated and saved. The disadvantage can be compensated by the fact that we can compare the current state of a network with all the combinations of interrupted links causing a break-up computed beforehand. In real time we can consequently obtain an alert if a worst-case scenario might occur in the network. We can also identify the links which have to be preserved as operational in order to avoid certain forms of traffic collapse. The links are not usually the same ones as in the worst-case scenarios but can still have a large impact on the network. The principal advantage of our algorithm is its deterministic nature. This means that it is able to precisely identify all the possible scenarios, unlike the stochastic approaches. The suggested approach can only be applied to network of a sufficient size. Despite the fact that it is able to identify all the break-ups, many combinations exist for larger networks and therefore there is also a certain limit related to computer performance. This limitation could be overcome using a stochastic approach.

We have further introduced a vulnerability measure based on the number of isolated people as a loss function which evaluates the impact of a given combination of interrupted links on the network (Results section). This approach shall be used during events which result in the disintegration of the network or in the phases of planning for the worst case scenarios. Additional measures evaluating the actual state of the network can be used as well. Several other vulnerability measures can also be used.

The main aim of the paper was to introduce a novel algorithm for computation of minimum k-cuts for a given number of interrupted links. This was the reason why we restricted our attention to the relatively simple vulnerability measure which can cover only several aspects of the impact of an event. It could be interesting in the next phase of the research to employ other vulnerability measures and analyze the disintegration of the network from the point of view of accessibility and connectivity.

This work adds to the current state of the art:

1. It introduces a new *deterministic* algorithm based upon the searching for the shortest cycles in order to identify all break-ups of a given network which are further evaluated by a vulnerability measure. It is demonstrated that this algorithm is much faster than any brute-force algorithm.
2. The algorithm represents an alternative approach to the lower and upper estimates of transportation network vulnerability ([54]) and spectral analysis ([5]). Compared to these papers, we are able to compute the precise value of a vulnerability measure and all the break-ups for a given number of interrupted links.
3. The algorithm is also able to provide results for large transportation networks corresponding to administrative units in reasonable time (compare to [20]).

Based on the arguments above, we believe that the incorporation of the algorithm into an online warning system as a tool for decision makers will have a significant positive impact on transportation security and could contribute to early warning before states of emergency.

## Supporting information

S1 File Data. An archive file with the program. It contains all the code files in java, libraries used and the Zlín region network files.
(ZIP)
